# Stabilizing HIV-1 envelope glycoprotein trimers to induce neutralizing antibodies

**DOI:** 10.1186/s12977-018-0445-y

**Published:** 2018-09-12

**Authors:** Alba Torrents de la Peña, Rogier W. Sanders

**Affiliations:** 1 Department of Medical Microbiology, Academic Medical Center, University of Amsterdam, 1105 AZ Amsterdam, The Netherlands; 2 Department of Microbiology and Immunology, Weill Medical College of Cornell University, New York, NY 10021 USA

## Abstract

An effective HIV-1 vaccine probably will need to be able to induce broadly neutralizing HIV-1 antibodies (bNAbs) in order to be efficacious. The many bNAbs that have been isolated from HIV-1 infected patients illustrate that the human immune system is able to elicit this type of antibodies. The elucidation of the structure of the HIV-1 envelope glycoprotein (Env) trimer has further fueled the search for Env immunogens that induce bNAbs, but while native Env trimer mimetics are often capable of inducing strain-specific neutralizing antibodies (NAbs) against the parental virus, they have not yet induced potent bNAb responses. To improve the performance of Env trimer immunogens, researchers have studied the immune responses that Env trimers have induced in animals; they have evaluated how to best use Env trimers in various immunization regimens; and they have engineered increasingly stabilized Env trimer variants. Here, we review the different approaches that have been used to increase the stability of HIV-1 Env trimer immunogens with the aim of improving the induction of NAbs. In particular, we draw parallels between the various approaches to stabilize Env trimers and ones that have been used by nature in extremophile microorganisms in order to survive in extreme environmental conditions.

## Background

The development of an effective and safe vaccine against HIV-1 requires a detailed understanding of the virological and immunological characteristics of HIV-1 infection. The virus has the ability to mutate very quickly, resulting in great viral diversity and making the development of an effective vaccine very challenging. Therefore, many research groups in the HIV-1 vaccine field pursue the development of a vaccine that can induce broadly neutralizing antibodies (bNAbs), i.e. antibodies that can target the functional envelope glycoprotein (Env) on many different virus isolates.

A focus of vaccine design is the generation of soluble Env trimer mimetics that can induce such antibodies and much progress has been made over the last few years in generating recombinant Env trimers that resemble the native Env spike. This required negating the inherent instability and flexibility of the native Env trimer and was accomplished by molecular design, resulting in soluble stable Env trimers, of which SOSIP.664 trimers were the prototype [1–4]. The clade A BG505 SOSIP.664 trimer, now the gold standard in HIV native-like trimer immunogen design, allowed the determination of the high-resolution structure of the Env trimer [5–7]. A recent structure of the membrane-derived JR-FL trimer confirmed that the soluble and stabilized BG505 trimer resembled the native Env trimer present on the viral membrane [8]. Moreover, the SOSIP.664 design could be extrapolated to HIV-1 isolates other than BG505, thereby expanding the toolkit for HIV-1 vaccine design [9–14]. When used as immunogens in animal trials, SOSIP.664 proteins from various strains elicited autologous (strain-specific) Tier-2 neutralizing antibodies (NAbs); however, these immunogens failed to elicit potent bNAbs in most animals [15–18].

Here, we describe several approaches that have been pursued in order to increase the performance of soluble Env trimer mimetics as immunogens to induce NAbs. First, we review different methods that have been used to improve the stability of HIV-1 Env trimers, including forced viral evolution, structure-based design, high throughput screening of mutant trimers and selection of improved trimers by mammalian cell display. We also review which epitopes on Env trimer mimetics are targeted by the immune system, and we assess different immunization strategies in which Env trimer immunogens can be employed, including cocktail and sequential vaccination regimens.

## Generating and validating mimetics of the native Env spike

The native Env trimer is unstable and flexible (conformationally heterogeneous), and the same applies to early generation soluble Env trimer derivates. As a consequence it took many years to elucidate its high-resolution structure by X-ray crystallography and cryo-electron microscopy (EM) techniques [19–21]. Initial low resolution cryo-electron tomography reconstructions of membrane-bound and soluble trimers provided new insights [22, 23], but high-resolution structures of the trimer were solved by using BG505 SOSIP.664 and the wide assortment of potent bNAbs that became available over the last decade [5, 24, 25]. Large gains in resolution were obtained with the first Env trimer crystal structure (4.7 Å resolution), which included a complex of the BG505 SOSIP.664 trimer with the V3-glycan bNAb PGT122 [20], and the first cryo-EM derived model of the same trimer in complex with the CD4 binding site bNAb PGV04 at a resolution of 5.8 Å [19]. In addition to providing lattice contacts to facilitate crystallization and 3D features to facilitate EM reconstruction, these bNAbs also provided validation of the structures, as the respective bNAb epitopes were clearly present.

The next step was to improve the resolution of the trimer structure by complexing the trimer with a combination of several new bNAbs. The use of the 35O22 bNAb directed to the gp120-gp41 interface and antibodies of the PGT121-family increased the resolution to ~ 3.5 Å and then 3.0 Å, and provided new details of the pre-fusion conformation of gp41, especially in HR1, a partially disordered region [6, 7, 26]. The SOSIP platform has been applied to trimers from different HIV-1 clades and their structures in complex with diverse bNAbs have also been elucidated, providing valuable new information for structure-based vaccine design [12, 21, 27–30]. Overall, the structures of all SOSIP trimers showed a highly similar trimer core, but revealed some differences in the variable loops that emanate from the core [21].

Another breakthrough came with the elucidation of the cryo-EM structure of a membrane-derived JR-FL trimer that was stabilized by the bNAb PGT151, but not by SOSIP mutations [8]. The overall structural features of the membrane-derived trimer as well as bNAb epitopes agree well to those of the soluble SOSIP trimers. However, subtle differences were observed in the HR1 region of gp41, where the I559P substitution in the soluble trimer breaks a helix that is present in the full-length Env structure, exactly as it was meant to do [1, 8]. The high similarity of the membrane-derived and the soluble version of the Env confirm the value of the SOSIP design for generating soluble Env spike mimetics. A modification of the SOSIP design involves the introduction of a flexible Gly-Ser linker between gp120 and gp41 to replace the furin cleavage site, sometimes with additional modifications, effectively resulting in single chains trimers that do not require furin cleavage [31–33].

## Designing next-generation Env trimers: learning from HIV-1 itself

A strategy to stabilize the Env trimers is by understanding and exploiting stability on the virus. To protect Env from NAbs, the virus evolves in a Darwinian way by selecting mutations in Env, in particular its variable loops, and by masking the protein surface with a shifting glycan shield. Virus evolution can also be exploited in the lab to obtain valuable information about mutations that can stabilize the Env trimer while retaining its functionality [34–37]. Such mutations can then be used to stabilize recombinant Env vaccine candidates.

**Fig. 1:**
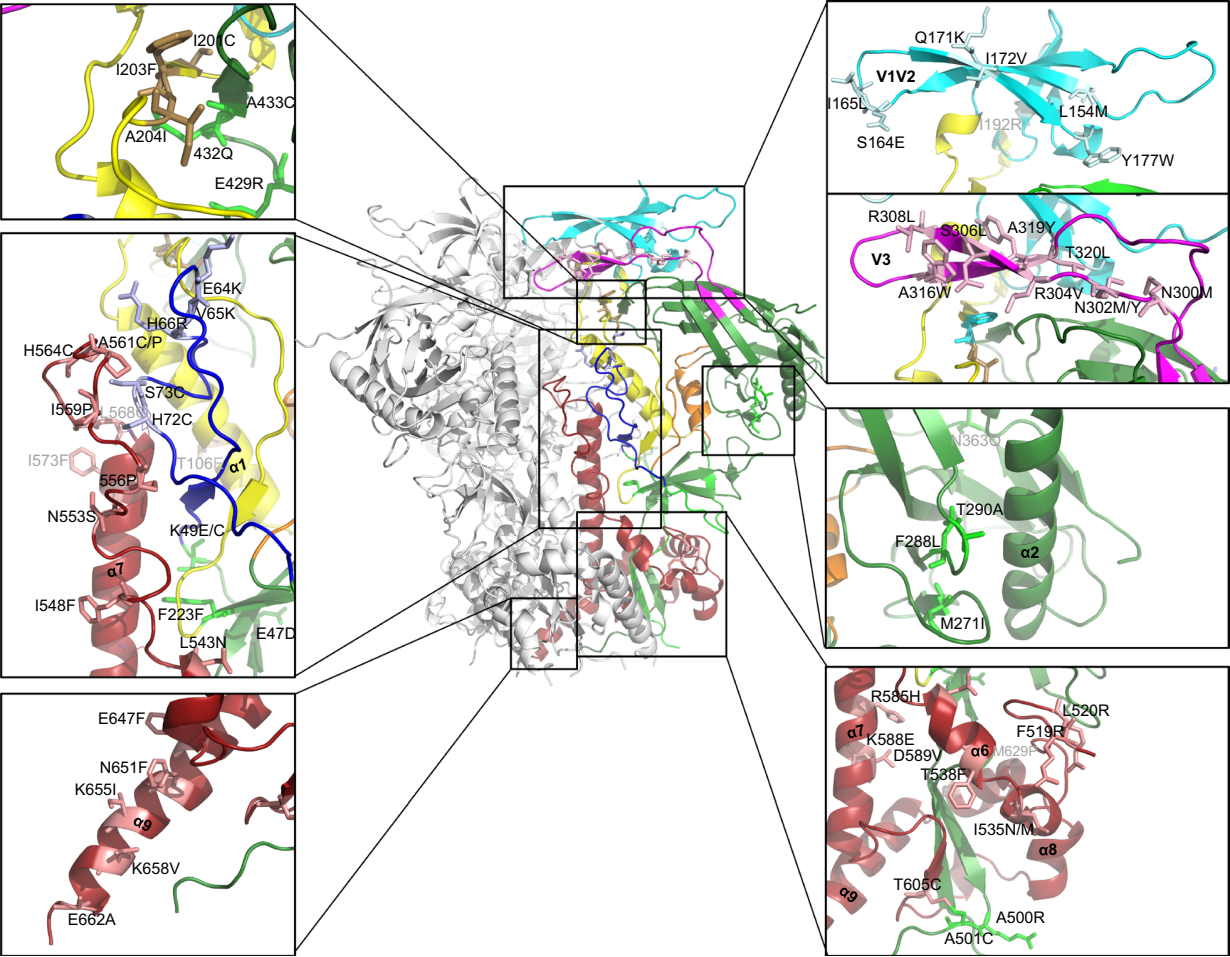
Amino acid substitutions that help stabilize soluble native-like trimers. Crystal structure of the BG505 SOSIP.664 trimer (5CEZ; [7]) displaying amino acid substitutions that stabilize native-like soluble trimers (see text for details). Two protomers are colored in white and one protomer is colored according to different regions. In gp120: V1V2 in cyan, V3 in magenta, inner domain layer 1 in blue, layer 2 in yellow, layer 3 in orange, N- and C-termini in green. Gp41 is colored in red. Boxes show detailed views of regions of the Env trimer that contain stabilizing amino acid substitutions. The substitutions were modeled by using the mutagenesis tool in Pymol molecular graphics system version 2.0.6 [102]

By culturing HIV-1 virus under harsh conditions such as unphysiological temperatures (45–55 °C) or incremental concentrations of denaturant (GuHCl), Leaman and colleagues identified a more stable Env mutant that contained seven mutations compared to its wild-type counterpart. Most of the mutations were located in the gp120-gp41 interface, including positions 535 and 543 (Fig. 1, Table 1) [34]. These substitutions were also identified by an earlier study in which the sequence of the early generation but relatively stable KNH1144 SOSIP protein was compared to that of the unstable JR-FL SOSIP [38]. De Taeye et al. introduced, when not present, the 535M and 543N mutations into distinct clade B (AMC008 and B41) and clade C trimers (ZM197M) in order to increase their trimerization and stability [10].

**Table 1:** Amino acid substitutions that stabilize soluble native-like trimers

	Hydrophobic	Aromatic	Proline/glycine	Disulfide bonds	Charged	Other
C1				E49C-L555C^a^	E47D^b^	E106T^b^
				H72C-H564C^a^	K49E^b^	
				A73C-A561C^a^	E64K^c^	
					V65K^b^	
					H66R^c^	
					T106E^d^	
V1	L154M^e^	Y177W^e^			S164E^b^	
V2	I165L^b^				Q171K^f^	
	I172V^f^				I192R^f^	
C2	A204I^g^	I203F^h^		I201C-A433C^m,n^		
	M271I^d^	F223W^d^				
	F288L^d^					
	T290A^d^					
V3	N300M^e^	N302Y/F^j^			H308R^b^	
	N302M^e^	A316W^c^				
	R304V^d^	A319Y^d^				
	S306L^i^					
	R308L^i^					
	T320L^e^					
C3						N363Q^d^
C4	Y420M^e^			I201C-A433C^m,n^	E429R^b^	R432Q^b^
C5				A501C-T605C^k^	A500R^b^	
FP^p^					F519R^j^	F516S^d^
					L520R^j^	
HR1^q^	D589V^g^	T538F^h^	L556P^g^	E49C-L555C^a^	L568D^d^	I535N/M^c,g,o^
		I548F^h^	I559G/P^l^	H72C-H564C^a^	K588E^g^	L543N^b,c^
		I573F^g^	A561P^d^	A73C-A561C^a^	G588R^b^	N553S^b^
			L568G^j^			V570H^d^
			T569G/P^j,l^			R585H^d^
						K588Q^g^
DSL^r^				A501C-T605C^k^		
	K655I^g^	E647F^g^	M629P^h^			
HR2^s^	K658V^g^	N651F^g^	S636G^j^			
	E662A^b^					

^a^Torrents de la Pena et al. Cell Rep. 2017

^b^Guenaga et al. J Virol. 2015

^c^de Taeye et al. Cell. 2015

^d^Steichen et al., Immunity. 2016

^e^Chuang et al. J Virol. 2017

^f^Ringe et al. J Virol. 2017

^g^Rutten et al. Cell Rep. 2018

^h^Sullivan et al. J Virol. 2017

^i^de Taeye et al. J Biol Chem. 2017

^j^Guenaga et al. Immunity. 2017

^k^Binley et al. J Virol. 2000

^l^Sanders et al. J Virol. 2002

^m^Guenaga et al. Plos Path. 2015

^n^Do Kwon et al., Nat. Struct. Mol. Biol. 2015

^o^Dey et al., Virol. 2007

^p^Fusion peptide

^q^Heptad repeat 1

^r^Disulfide loop

^s^Heptad repeat 2

Other substitutions that can improve native-like trimers were selected based on studies on how the virus becomes dependent on the entry inhibitor VIR165, and how HIV-1 can adapt to cold [39, 40]. These substitutions are located in C1 domain of gp120 (E64K, H66R and H66A; Fig. 1, Table 1) and likely keep the virus in the prefusion conformation by impeding steps towards the CD4-bound conformation by interacting with the HR1 region in gp41 [10, 41]. Thus, mutations that increase the stability of the native Env spike on virions can also be useful for the development of stable soluble native-like Env immunogens.

## Designing next-generation Env trimers: learning from extremophile organisms

SOSIP trimers based on most virus isolates other than BG505 initially did not form stable native-like trimers efficiently. However, the available trimer structures provided sufficient structural details to design modifications that improve the structure and stability of Env trimers, and that allowed generating stable trimers from many different isolates and clades.

When considering how to stabilize vaccine antigens, much can be learnt from nature. Extremophile bacteria and archea, which thrive in extreme environmental conditions such as high and low temperatures (between 45–122 °C and below − 15 °C, respectively) or alkaline and acidic conditions (pH > 11 and pH < 1, respectively) [42–44], have evolved highly stable proteins compared to their mesophilic homologues [43, 45]. In extremophile organisms, natural evolution has applied six methods of protein stabilization. Several of these methods have been applied, either intentionally or not, to HIV-1 Env trimer vaccine design.

First, thermophilic proteins often have an increased number of hydrophobic residues at domain and oligomer interfaces, facilitating tighter packing of protein domains [46, 47]. A similar strategy was applied to HIV-1 Env trimers to stabilize the trimer and prevent the exposure of non-NAbs [48–53]. For example, de Taeye et al. avoided the spontaneous exposure of the V3 loop by increasing the hydrophobic interactions within the V3 domain and between the V3 and V1V2 domains, by introducing two Leu residues (S306L, R308L) in the V3 loop (Fig. 1, Table 1) [53]. Similarly, Chuang et al., Kulp et al., Steichen et al. and Rutten et al., introduced hydrophobic mutations in the trimer core (A204I, T320L, E381M, Q422L) or the trimer stem (D589V, K655I, K658V, E662A) using structure-based design and mammalian cell display, which resulted in increased Env packing and reduced flexibility (Fig. 1, Table 1) [49–51, 54].

Second, extremophile proteins contain a higher number of aromatic amino acids, which can enhance protein thermostability through ring stacking interactions as well as hydrophobic packing [55–57]. In structure-based HIV-1 immunogen design, several groups used the same principle and introduced aromatic residues to reduce V3 exposure (A316W, A319Y), and to increase stability of the trimer apex (Y177W, N302Y, N302F), the trimer base (E647F, N651F) and the trimer interface (gp120-gp41 interface: A223W, T538F and I548F; gp41-gp41 interface: I573F) (Fig. 1, Table 1) [10, 48–51, 54]. Overall, the introduction of hydrophobic and aromatic residues accounts for ~ 45% of the total number of mutations that are described in the literature to increase Env trimer stability.

Third, proteins from thermophilic organisms tend to have an increased number of charged residues involved in internal ion pairing and hydrogen bonding, as well as an increased number of positively charged residues at the solvent-exposed surface to provide stability at the surface [57]. For HIV-1 Env trimers the introduction of charged amino acids at the gp120 and gp41 interface also contributed to formation of well-ordered native-like trimers from different clades with enhanced thermostability (A500R, A558R) (Fig. 1, Table 1) [13, 58].

Fourth, proteins from thermophilic organisms usually contain many more predicted disulfide bonds than mesophilic organisms, which increases protein stability dramatically [45, 59, 60]. In mesophiles, proteins with many disulfide bonds are rare. As a consequence, there is a strong positive correlation between the number of disulfide bonds in proteins and the maximum growth temperature of thermophilic organisms [45, 59, 60]. Some viruses, such as influenza and vaccinia viruses, contain a disulfide bond that links the two Env subunits together, but HIV-1 Env naturally does not have such a disulfide bond, resulting in shedding of the gp120 subunit. The first step of generating stable native-like trimers was therefore the introduction of a disulfide bond between the gp120 and gp41 subunits (A501C-T605C) (Fig. 1, Table 1) [2]. To stabilize the flexible trimer interface, additional disulfide bonds have been introduced in the Env trimer: an intersubunit disulfide bond (A73C-A561C) and an interprotomer disulfide bond (E49C-L555C) (Fig. 1, Table 1) [7, 61]. Furthermore, an intrasubunit disulfide bond (I201C-A433C) described by Kwon et al. and Guenaga et al. also stabilized the trimer in its pre-fusion state (Fig. 1, Table 1) [62, 63]. Combining three non-native disulfide bonds (A501C-T605C + A73C-A561C + I201C-A433C or A501C-T605C + A73C-A561C + E49C-L555C) resulted in hyperstable trimers that reached melting temperatures of up to 81 °C and 92 °C, respectively [61].

Fifth, thermophilic organisms increase the number of proline and glycine residues in loops to provide conformational rigidity to the protein [43]. In the HIV field, similar approaches have been used to generate soluble Env trimers. Since the HR1 region forms a helix in the post-fusion state and it adopts a partially disordered conformation in the pre-fusion state, we introduced the I559P mutation in the loop of HR1 to destabilize the post-fusion state of gp41 and stabilize the pre-fusion state [1]. Similarly, the introduction of glycine or proline residues in the HR1 and HR2 (N554G, L556P, A558P, I559G, T569P, T569G and S636G) further stabilized soluble HIV-1 Env trimers (Fig. 1, Table 1) [1, 54, 58]. Kong et al. computationally modeled HR1 loops with low Gibbs free energy that resulted in increased numbers of proline residues and rigidification of the HR1 loop [64].

A last mechanism that thermophilic organisms apply to survive at high temperatures is the reduction of asparagine and glutamine residues to prevent deamidation. This strategy has not been (intentionally) used for HIV vaccine design yet.

Thus, strategies to stabilize Env trimers from BG505 and other isolates using high throughput screening, selection by mammalian display, and structure-based design, in many ways mirror what extremophiles have achieved in nature to survive under extreme conditions. The resulting improvements in stability of soluble Env trimers allow us to use these immunogens in immunogenicity studies by facilitating the generation of a toolkit of trimers from different clades. Several of these trimers have been evaluated as immunogens and some studies have suggested that in some cases increased thermostability translates into increased immunogenicity [61, 65]. Furthermore, by increasing the trimer shelf life and avoiding cold chain transportation and storage will help to eventually produce a vaccine that is globally available.

## Evaluating Env trimers in vivo: learning from immunization experiments

Native-like Env trimers have been tested as immunogens in small animals, mostly rabbits, and nonhuman primates. These studies indicated that native-like trimers consistently induced, for the first time, NAb responses against hard-to-neutralize (Tier 2) primary HIV-1 isolates. However, heterologous primary isolates were not, or only weakly and sporadically neutralized. Highly stable native-like trimers have been designed to improve the immunogenicity of the trimer by increasing its half-life in vivo and thus the presentation of bNAb epitopes. Immunogenicity studies with the highly stable trimers did not increase the generation of autologous NAb responses, but they induced weak heterologous Tier 2 responses in some cases. While trimer thermostability in vitro is a useful parameter that can be linked to in vivo observations [61, 65], it will also be important to investigate additional stability parameters such as trimer stability in serum at 37 °C.

Immunization with SOSIP trimers also induced strong non-neutralizing antibody (non-NAb) responses against V3 epitopes and neo-epitopes at the bottom of the trimer [10, 15, 16, 66]. Heterologous primary isolates were not, or only weakly and sporadically neutralized, pointing to possible directions of further research to improve native-like trimer immunogens.

First, it has recently been shown that the NAb responses in animals immunized with BG505 SOSIP trimers are dominated by specificities targeting a hole in the glycan shield, specifically the peptidic surface surrounding amino acids at positions 241 and 289, where most virus isolates have N-linked glycans [17, 67]. While autologous NAb responses might in some cases be a starting point for generating bNAb responses [7, 68], they could also distract or compete for such responses. If the latter scenario were true, one might want to dampen immunodominant isolate-specific, glycan-hole directed NAb responses. One strategy to counteract the immunogenicity of the BG505 specific glycan hole would be to immunize with trimers that contain glycans at positions N241 and N289. Previous studies have shown that immunizations with trimers based on isolates with a denser glycan shield (AMC008 and ZM197M) induced a broader heterologous NAb response compared to trimers from isolates with large holes in the glycan shield (BG505 and B41), which supports the pursuit of this strategy [69].

Second, immunization with BG505 SOSIP.664 trimers induced a strong response against non-NAb V3 epitopes [10, 50, 53, 70], leading to the hypothesis that this immunodominant V3-response interfered with the generation of bNAb responses. When rabbits were immunized with an improved version of the trimer, the BG505 SOSIP.v4 trimer, which contained the A316W mutation that sequestered the V3 epitope, these SOSIP trimers induced weaker anti-V3 responses and V3-directed Tier 1A virus NAb responses, without affecting the autologous NAb response [10, 16]. In a next iteration of trimer design, two additional hydrophobic residues were incorporated in the V3 loop of the BG505 SOSIP.v4 trimer (R306L and R308L) to completely abolish the responses against the V3 loop [53]. Although these modifications reduced V3 immunogenicity, they did not improve the autologous NAb responses, nor did they result in a broadening of the NAb response. Similar results were recently obtained by Kulp et al. using different V3 designs [16, 50].

Third, the generation of soluble Env trimers resulted in the exposure of neo-epitopes at the bottom of the trimer, which is occluded by the viral membrane when the Env trimer is presented on virions. It has been suggested that the bottom of the trimer presents another immunodominant non-NAb epitope that could interfere with NAb responses [66, 70]; M. J. van Gils, C. A. Cottrell, A. B. Ward, R. W. Sanders unpublished data). To prevent the exposure of this epitope one could hide it, for example by placing the trimer on a nanoparticle.

Although interference by V3 and trimer bottom non-NAb responses is an attractive hypothesis, there is no formal proof yet that these non-NAb responses interfere with more desirable NAb and bNAb reponses. However, the V3 and trimer bottom non-NAb epitopes are usually solely of peptidic nature. B cells recognizing such epitopes are much more frequent in the naïve B cell repertoire and probably have higher affinity than naïve B cells recognizing composite peptide-glycan bNAb epitopes [70]. Higher affinity B cells might have a selective advantage over the lower affinity B cells targeting bNAb epitopes, because they might bind and process more antigen and, as a consequence, receive more T cell help. This will make it unlikely that B cells with the intrinsic capacity to mature into bNAbs will thrive in an environment that favors B cells targeting non-NAb or strain-specific glycan hole NAb epitopes. However, these arguments are somewhat theoretical in the HIV-1 context and the immune responses raised against Env trimers in animal and human vaccination experiments should be dissected in more detail to address these concerns.

To improve our understanding of the fate of Env trimers in vivo a number of studies focused on the germinal center responses against Env trimers. Macaques were immunized with stable Env trimers and germinal center cells from the lymph nodes were collected over time using fine needle aspirates (FNA), thereby avoiding the need to take lymph node biopsies and thereby blunting the response in that lymph node [18, 70]. While all the macaques generated immune responses against the trimer, the NAb responses correlated quantitatively with GC B cell frequencies. These studies provide a frame-of-reference for further studies on germinal center B cells and Tfh cells and their roles in epitope immunodominance and subdominance. Furthermore, insights in the amount of Env that enters the lymph nodes and the half-life of the Env protein in circulation would help efforts to study how the immunogen is delivered to B cells and how this can be improved. Previous work on other immunogens, including on gp120, suggest that it is worthwhile to exploit fluorescently tagged native-like trimers and to answer some of these questions, especially whether highly stable trimers show longer trimer half life in the presence of serum and proteases [71–73].

## Evaluating Env trimers in vivo: learning from different immunization regimens

Until now, monovalent immunization with soluble HIV-1 Env trimers has only induced strong NAb responses against autologous viruses, and only weak and sporadic heterologous Tier 2 NAb responses. One strategy to increase neutralization breadth involves exploring different vaccine regimens such as cocktails of different immunogens. HIV-1 is a highly diverse pathogen, as is influenza virus. For influenza virus we use annually updated vaccines composed of a trivalent or tetravalent cocktail of different inactivated influenza viruses. However, annual influenza vaccination only protects against viral variants that are closely related to the vaccine strains, which exemplifies how difficult it is to induce a bNAb response against highly diverse viruses. The search for a universal flu vaccine shares similarities with the search for a bNAb-inducing HIV-1 vaccine.

To increase neutralization breadth, we have explored the use of cocktail and sequential regimens [17, 69]. We observed that immunization with a combination of immunogens in a cocktail formulation or in sequence did not induce bNAbs, but merely autologous NAb responses. Furthermore, the autologous NAb responses were prominent against the most immundominant trimer of the cocktail [69]. Thus, the immune response shows narrow specificity, similar to what has been reported for influenza vaccines [74]. These results indicate that an HIV-1 Env vaccine based on a cocktail or sequence of randomly chosen trimers is unlikely to induce bNAbs.

An alternative to the cocktail and sequential formulations could be to guide naïve B cell lineages towards bNAb activity by rational design. Since in natural infection the bNAbs develop through the co-evolution of the virus and the antibodies, one strategy that is being pursued is immunization with longitudinal Env sequences from patients that developed a bNAb response [75–80]. This strategy aims to recapitulate the evolutionary path of the virus and assumes that the development of the bNAb response largely depends on viral characteristics. Another, but somewhat related strategy, termed germline-targeting, focuses on the activation of rare subsets of naïve B cell that express B cell receptors (germline precursors) that have the intrinsic capacity to develop into bNAbs. SOSIP trimers generally do not bind inferred germline versions of bNAbs and several groups are designing immunogens that bind specifically to germline antibodies to guide the B cell responses towards the development of broadly neutralizing antibodies [51, 81–87].

Trimers can also be used to boost responses that are primed by epitope-specific immunogens. For example, Xu et al. applied trimers in an immunization regimen aimed at focusing immune responses to the fusion peptide. They immunized guinea pigs and macaques with a fusion peptide coupled to the carrier protein KLH, and boosted the responses with stabilized BG505 SOSIP trimers. This immunization strategy induced autologous NAb responses in all the animals and substantial NAb responses against heterologous Tier-2 viruses in some animals [88]. When they isolated the antibodies that were responsible for the broad neutralization they could confirm that these antibodies targeted the fusion peptide on both autologous and heterologous viruses [88].

Another strategy to overcome the low affinity of the immunogens to the desired but rare germline precursors of bNAbs is to multimerize the antigen, thereby increasing the potency of the Ab response by cross-linking the B cell receptors. The use of liposomes and ferritin nanocages that present Env trimers on their surface indeed improve the NAb response [89–91]. The flexibility of the nanoparticle system would allow the incorporation of trimers from different clades or lineages to enhance NAb responses against conserved B cell epitopes.

## Applying the lessons learnt to other viral pathogens

We described how to make stable HIV-1 Env trimers for structural and immunological studies and how to use them in the quest for an HIV-1 vaccine. However, the lessons learnt in the HIV-1 field can also be applied to other viruses and *vice versa*. Similar to HIV-1 Env, other viral fusion proteins, such as the respiratory syncytial virus (RSV) F protein, are intrinsically metastable and easily switch from the pre-fusion to the post-fusion form. While a lot of efforts had to be invested to produce a stable soluble HIV-1 Env trimer, the influenza HA protein is comparatively stable and can be easily expressed. In contrast, the RSV F protein is, similarly to HIV Env, quite unstable and it adopts the post-fusion conformation when purified as soluble protein. While McLellan and colleagues introduced a disulfide bond and hydrophobic residues to keep the RSV glycoprotein in the pre-fusion state [92], Krarup et al. prevented the transition of this protein to the postfusion state by introducing helix-breaking prolines in the refolding region 1, quite similar to what has been done for HIV-1 Env [93].

Recently, high-resolution structures of other viral glycoproteins were solved, including those of human parainfluenza virus 5, ebola virus, lassa virus, human betacoronavirus HKS1, lymphocytic choriomeningitis virus, herpes simplex virus 1 and severe fever with thrombocytopenia syndrome virus [92, 94–100]. The above-mentioned strategies that worked for HIV-1 Env have also benefited the stabilization and native-like pre-fusion forms of several of these glycoproteins. To keep the Middle East respiratory syndrome coronavirus (MERS-CoV) glycoprotein in the pre-fusion state, Pallesen et al. introduced two prolines at the start of the central helix of the protein, similarly to the I559P substitution introduced in the HIV-1 Env trimer [1, 96]. Similarly, to retain the lassa virus glycoprotein in the pre-fusion conformation, Hastie and colleagues incorporated a proline in the HR1 domain [98]. To further improve the stability the authors introduced a disulfide bond between the two subunits and improved the cleavage site as previously done for the HIV-1 Env trimer. Thus, the general strategy is to retain the viral glycoprotein in the pre-fusion conformation by structure-based design [2, 92, 96].

To further improve the immunogenicity of Env trimers, we can also learn from the recombinant vaccines against viral pathogens that are currently available. Hepatitis B virus, hepatitis E virus and human papillomavirus use recombinant virus-like particles as the immunogen [101]. These vaccines are self-assembling nanoparticles that mimic the native virions and expose neutralizing epitopes on their surface. As previously discussed, improvement of the nanoparticle design in the HIV-1 vaccine field is being pursued by several groups including us. In short, the strategies used to improve HIV-1 immunogen design provide a template to design vaccine candidates for other viruses and *vice versa.*

## Conclusion

Here, we reviewed the latest design strategies to stabilize the soluble HIV-1 Env trimers as well as different immunization strategies maximize their value. The development of native-like trimers as immunogens, the availability of high-resolution structures, the design of different immunization strategies, the promise of germline-targeting and nanoparticle presentation, combined with an increased understanding of the host immunological responses against Env trimers, should advance the field of HIV-1 trimer vaccinology. These efforts should advance the HIV-1 field and provide lessons for subunit vaccines against other viruses for which diversity is an issue, such as, but not limited to, influenza virus, dengue virus and hepatitis C virus.

## Abbreviations

bNAbs: Broadly neutralizing antibodies
Env: Envelope glycoprotein
NAbs: Neutralizing antibodies
EM: Electron microscopy
non-NAb: Non-neutralizing antibody
FNA: Fine needle aspirates
RSV: Respiratory syncytial virus
MERS-CoV: Middle East respiratory syndrome coronavirus

## References

[1] Sanders RW, Vesanen M, Schuelke N, Master A, Schiffner L, Kalyanaraman R (2002). Stabilization of the soluble, cleaved, trimeric form of the envelope glycoprotein complex of human immunodeficiency virus type 1. J Virol.

[2] Binley JM, Sanders RW, Clas B, Schuelke N, Master A, Guo Y (2000). A recombinant human immunodeficiency virus type 1 envelope glycoprotein complex stabilized by an intermolecular disulfide bond between the gp120 and gp41 subunits is an antigenic mimic of the trimeric virion-associated structure. J Virol.

[3] Khayat R, Lee JH, Julien J-P, Cupo A, Klasse PJ, Sanders RW (2013). Structural characterization of cleaved, soluble HIV-1 envelope glycoprotein trimers. J Virol.

[4] Klasse PJ, Depetris RS, Pejchal R, Julien J-P, Khayat R, Lee JH (2013). Influences on trimerization and aggregation of soluble, cleaved HIV-1 SOSIP envelope glycoprotein. J Virol.

[5] Sanders RW, Derking R, Cupo A, Julien JP, Yasmeen A, de Val N (2013). A next-generation cleaved, soluble HIV-1 Env trimer, BG505 SOSIP.664 gp140, expresses multiple epitopes for broadly neutralizing but not non-neutralizing antibodies. PLoS Pathog.

[6] Pancera M, Zhou T, Druz A, Georgiev IS, Soto C, Gorman J (2014). Structure and immune recognition of trimeric pre-fusion HIV-1 Env. Nature.

[7] Garces F, Lee JHH, de Val N, Torrents de la Peña A, Kong L, Puchades C (2015). Affinity maturation of a potent family of HIV antibodies is primarily focused on accommodating or avoiding glycans. Immunity.

[8] Lee JH, Ozorowski G, Ward AB (2016). Cryo-EM structure of a native, fully glycosylated, cleaved HIV-1 envelope trimer. Science.

[9] Pugach P, Ozorowski G, Cupo A, Ringe R, Yasmeen A, de Val N (2015). A native-like SOSIP.664 trimer based on an hiv-1 subtype B *env* gene. J Virol.

[10] de Taeye SW, Ozorowski G, Torrents de la Peña A, Guttman M, Julien JP, van den Kerkhof TLGM (2015). Immunogenicity of stabilized HIV-1 envelope trimers with reduced exposure of non-neutralizing epitopes. Cell.

[11] Julien J, Lee JH, Ozorowski G, Hua Y, Torrents de la Peña A, de Taeye SW (2015). Design and structure of two HIV-1 clade C SOSIP.664 trimers that increase the arsenal of native-like Env immunogens. Proc Natl Acad Sci USA.

[12] Stewart-Jones GBE, Soto C, Lemmin T, Chuang GY, Druz A, Kong R (2016). Trimeric HIV-1-Env structures define glycan shields from clades A, B, and G. Cell.

[13] Guenaga J, de Val N, Tran K, Feng Y, Satchwell K, Ward AB (2015). Well-ordered trimeric HIV-1 subtype B and C soluble spike mimetics generated by negative selection display native-like properties. PLoS Pathog.

[14] Ringe RP, Yasmeen A, Ozorowski G, Go EP, Pritchard LK, Guttman M (2015). Influences on the design and purification of soluble, recombinant native-like HIV-1 envelope glycoprotein trimers. J Virol.

[15] Sanders RW, van Gils MJ, Derking R, Sok D, Ketas TJ, Burger JA (2015). HIV-1 neutralizing antibodies induced by native-like envelope trimers. Science..

[16] Pauthner M, Havenar-Daughton C, Sok D, Nkolola JP, Bastidas R, Boopathy AV (2017). elicitation of robust Tier 2 neutralizing antibody responses in nonhuman primates by HIV envelope trimer immunization using optimized approaches. Immunity.

[17] Klasse PJ, LaBranche CC, Ketas TJ, Ozorowski G, Cupo A, Pugach P (2016). Sequential and simultaneous immunization of rabbits with HIV-1 envelope glycoprotein SOSIP.664 trimers from clades A, B and C. PLoS Pathog.

[18] Havenar-Daughton C, Reiss SM, Carnathan DG, Wu JE, Kendric K, Torrents de la Peña A (2016). Cytokine-independent detection of antigen-specific germinal center T follicular helper cells in immunized nonhuman primates using a live cell activation-induced marker technique. J Immunol.

[19] Lyumkis D, Julien J-P, de Val N, Cupo A, Potter CS, Klasse P-J (2013). Cryo-EM structure of a fully glycosylated soluble cleaved HIV-1 envelope trimer. Science.

[20] Julien J-P, Cupo A, Sok D, Stanfield RL, Lyumkis D, Deller MC (2013). Crystal structure of a soluble cleaved HIV-1 envelope trimer. Science.

[21] Ward AB, Wilson IA (2017). The HIV-1 envelope glycoprotein structure: nailing down a moving target. Immunol Rev.

[22] Harris A, Borgnia MJ, Shi D, Bartesaghi A, He H, Pejchal R (2011). Trimeric HIV-1 glycoprotein gp140 immunogens and native HIV-1 envelope glycoproteins display the same closed and open quaternary molecular architectures. Proc Natl Acad Sci USA.

[23] Liu J, Bartesaghi A, Borgnia MJ, Sapiro G, Subramaniam S (2008). Molecular architecture of native HIV-1 gp120 trimers. Nature.

[24] Wu X, Parast AB, Richardson BA, Nduati R, John-stewart G, Mbori-ngacha D (2006). Neutralization escape variants of human immunodeficiency virus type 1 are transmitted from mother to infant. J Virol..

[25] McCoy LE, Burton DR (2017). Identification and specificity of broadly neutralizing antibodies against HIV. Immunol Rev.

[26] Doria-Rose N, Schramm C, Gorman J, Moore PL, Bhiman JN, DeKosky BJ (2014). Developmental pathway for potent V1V2-directed HIV-neutralizing antibodies. Nature.

[27] Jardine JG, Sok D, Julien JP, Briney B, Sarkar A, Liang CH (2016). Minimally mutated HIV-1 broadly neutralizing antibodies to guide reductionist vaccine design. PLoS Pathog.

[28] Kong L, Torrents De La Peña A, Deller MC, Garces F, Sliepen K, Hua Y (2015). Complete epitopes for vaccine design derived from a crystal structure of the broadly neutralizing antibodies PGT128 and 8ANC195 in complex with an HIV-1 Env trimer. Acta Crystallogr Sect D: Biol Crystallogr.

[29] Scharf L, Wang H, Gao H, Chen S, McDowall AW, Bjorkman PJ (2015). Broadly neutralizing antibody 8ANC195 recognizes closed and open states of HIV-1 Env. Cell.

[30] Lee JH, Andrabi R, Su CY, Yasmeen A, Julien JP, Kong L (2017). A broadly neutralizing antibody targets the dynamic HIV envelope trimer apex via a long, rigidified, and anionic β-hairpin structure. Immunity.

[31] Georgiev IS, Joyce MG, Yang Y, Sastry M, Zhang B, Baxa U (2015). Single-chain soluble BG505.SOSIP gp140 trimers as structural and antigenic mimics of mature closed HIV-1 Env. J Virol.

[32] Sharma SK, deVal N, Bale S, Guenaga J, Tran K, Feng Y (2015). Cleavage-independent HIV-1 Env trimers engineered as soluble native spike mimetics for vaccine design. Cell Rep.

[33] Kong L, He L, De Val N, Vora N, Morris CD, Azadnia P (2016). Uncleaved prefusion-optimized gp140 trimers derived from analysis of HIV-1 envelope metastability. Nat Commun.

[34] Leaman DP, Zwick MB (2013). Increased functional stability and homogeneity of viral envelope spikes through directed evolution. PLoS Pathog.

[35] Agrawal N, Leaman DP, Rowcliffe E, Kinkead H, Nohria R, Akagi J (2011). Functional stability of unliganded envelope glycoprotein spikes among isolates of human immunodeficiency virus type 1 (HIV-1). PLoS ONE.

[36] Bontjer I, Land A, Eggink D, Verkade E, Tuin K, Baldwin C (2009). Optimization of human immunodeficiency virus type 1 envelope glycoproteins with V1/V2 deleted, using virus evolution. J Virol.

[37] Bontjer I, Melchers M, Eggink D, David K, Moore JP, Berkhout B (2010). Stabilized HIV-1 envelope glycoprotein trimers lacking the V1V2 domain, obtained by virus evolution. J Biol Chem.

[38] Dey AK, David KB, Klasse PJ, Moore JP (2007). Specific amino acids in the N-terminus of the gp41 ectodomain contribute to the stabilization of a soluble, cleaved gp140 envelope glycoprotein from human immunodeficiency virus type 1. Virology.

[39] Eggink D, de Taeye SW, Bontjer I, Klasse PJ, Langedijk JPM, Berkhout B (2016). HIV-1 escape from a peptidic anchor inhibitor by envelope glycoprotein spike stabilization. J Virol.

[40] Kassa A, Finzi A, Pancera M, Courter JR, Smith AB, Sodroski J (2009). Identification of a human immunodeficiency virus type 1 envelope glycoprotein variant resistant to cold inactivation. J Virol.

[41] Finzi A, Xiang SH, Pacheco B, Wang L, Haight J, Kassa A (2010). Topological layers in the HIV-1 gp120 inner domain regulate gp41 interaction and CD4-triggered conformational transitions. Mol Cell.

[42] D’Amico S, Gerday C, Feller G (2001). structural determinants of cold adaptation and stability in a large protein. J Biol Chem.

[43] Redd JC, Lewis H, Trejo E, Winston V, Evilia C (2013). Protein adaptations in archael extremophiles. Archaea.

[44] del Vecchio P, Elias M, Merone L, Graziano G, Dupuy J, Mandrich L (2009). Structural determinants of the high thermal stability of SsoPox from the hyperthermophilic archaeon Sulfolobus solfataricus. Extremophiles.

[45] Liszka MJ, Clark ME, Schneider E, Clark DS (2012). Nature versus nurture: developing enzymes that function under extreme conditions. Annu Rev Chem Biomol Eng.

[46] Melchionna S, Sinibaldi R, Briganti G (2006). Explanation of the stability of thermophilic proteins based on unique micromorphology. Biophys J.

[47] Razvi A, Scholtz JM (2006). Lessons in stability from thermophilic proteins. Protein Sci.

[48] Sullivan JT, Sulli C, Nilo A, Yasmeen A, Ozorowski G, Sanders RW (2017). High-throughput protein engineering improves the antigenicity and stability of soluble HIV-1 envelope glycoprotein SOSIP trimers. J Virol.

[49] Chuang G-Y, Gneg H, Pancera M, Xu K, Cheng C, Acharya P (2017). Structure-based design of a soluble prefusion-closed HIV-1 Env trimer with reduced CD4 affinity and improved immunogenicity. J Virol.

[50] Kulp DW, Steichen JM, Pauthner M, Hu X, Schiffner T, Liguori A (2017). Structure-based design of native-like HIV-1 envelope trimers to silence non-neutralizing epitopes and eliminate CD4 binding. Nat Commun.

[51] Steichen JM, Kulp DW, Tokatlian T, Escolano A, Dosenovic P, Stanfield RL (2016). HIV vaccine design to target germline precursors of glycan-dependent broadly neutralizing antibodies. Immunity.

[52] de Taeye SW, Moore JP, Sanders RW (2016). HIV-1 Envelope trimer design and immunization strategies to induce broadly neutralizing antibodies. Trends Immunol.

[53] de Taeye SW, Torrents de la Peña A, Vecchione A, Scutigliani E, Sliepen K, Burger JA (2017). Stabilization of the gp120 V3 loop through hydrophobic interactions reduces the immunodominant V3-directed non-neutralizing response to HIV-1 envelope trimers. J Biol Chem.

[54] Rutten L, Lai YT, Blokland S, Truan D, Bisschop IJM, Strokappe NM (2018). A universal approach to optimize the folding and stability of prefusion-closed HIV-1 envelope trimers. Cell Rep.

[55] Goldstein RA (2007). Amino-acid interactions in psychrophiles, mesophiles, thermophiles, and hyperthermophiles: insights from the quasi-chemical approximation. Protein Sci.

[56] Makwana KM, Mahalakshmi R (2015). Implications of aromatic-aromatic interactions: from protein structures to peptide models. Protein Sci.

[57] Zhou XX, Wang YB, Pan YJ, Li WF (2008). Differences in amino acids composition and coupling patterns between mesophilic and thermophilic proteins. Amino Acids.

[58] Guenaga J, Garces F, De Val N, Ward AB, Wilson IA, Wyatt RT (2017). Glycine substitution at helix-to-coil transitions facilitates the structural determination of a stabilized subtype c hiv envelope glycoprotein. Immunity.

[59] Beeby M, O’Connor BD, Ryttersgaard C, Boutz DR, Perry LJ, Yeates TO (2005). The genomics of disulfide bonding and protein stabilization in thermophiles. PLoS Biol.

[60] Ladenstein R, Ren B (2008). Reconsideration of an early dogma, saying “there is no evidence for disulfide bonds in proteins from archaea”. Extremophiles.

[61] Torrents de la Peña A, Julien J-P, De Taeye SW, Ward AB, Wilson IA, Sanders RW (2017). Improving the immunogenicity of native-like HIV-1 envelope trimers by hyperstabilization. Cell Rep..

[62] Do Kwon Y, Pancera M, Acharya P, Georgiev IS, Crooks ET, Gorman J (2015). Crystal structure, conformational fixation and entry-related interactions of mature ligand-free HIV-1 Env. Nat Struct Mol Biol.

[63] Guenaga J, Dubrovskaya V, de Val N, Sharma SK, Carrette B, Ward AB (2016). Structure-guided redesign increases the propensity of HIV Env to generate highly stable soluble trimers. J Virol.

[64] Kong L, He L, De Val N, Vora N, Morris CD, Azadnia P (2016). Uncleaved prefusion-optimized gp140 trimers derived from analysis of HIV-1 envelope metastability. Nat Commun.

[65] Feng Y, Tran K, Bale S, Kumar S, Guenaga J, Wilson R (2016). Thermostability of well-ordered HIV spikes correlates with the elicitation of autologous Tier 2 neutralizing antibodies. PLoS Pathog.

[66] Hu JK, Crampton JC, Cupo A, Ketas T, van Gils MJ, Sliepen K (2015). Murine antibody responses to cleaved soluble HIV-1 envelope trimers are highly restricted in specificity. J Virol.

[67] McCoy LE, van Gils MJ, Ozorowski G, Messmer T, Briney B, Voss JE (2016). Holes in the glycan shield of the native hiv envelope are a target of trimer-elicited neutralizing antibodies. Cell Rep.

[68] Lynch RM, Wong P, Tran L, O’Dell S, Nason MC, Li Y (2015). HIV-1 Fitness cost associated with escape from the VRC01 class of CD4 binding site neutralizing antibodies. J Virol.

[69] Torrents de la Peña A, de Taeye SW, Sliepen K, LaBranche C, Burger JA, Schermer EE (2018). Immunogenicity in rabbits of SOSIP trimers from clades A, B and C given individually, sequentially or in combinations. J Virol.

[70] Havenar-Daughton C, Lee JH, Crotty S (2017). Tfh cells and HIV bnAbs, an immunodominance model of the HIV neutralizing antibody generation problem. Immunol Rev.

[71] Park C, Arthos J, Cicala C, Kehrl JH (2015). The HIV-1 envelope protein gp120 is captured and displayed for B cell recognition by SIGN-R1 + lymph node macrophages. Elife.

[72] Sliepen K, Van Montfort T, Ozorowski G, Pritchard LK, Crispin M, Ward AB (2015). Engineering and characterization of a fluorescent native-like HIV-1 envelope glycoprotein trimer. Biomolecules.

[73] Forthal DN, Gilbert PB, Landucci G, Phan T (2007). Recombinant gp120 vaccine-induced antibodies inhibit clinical strains of HIV-1 in the presence of Fc receptor-bearing effector cells and correlate inversely with HIV infection rate. J Immunol.

[74] Gerdil C (2003). The annual production cycle for influenza vaccine. Vaccine.

[75] Bonsignori M, Liao H-X, Gao F, Williams WB, Alam SM, Montefiori DC (2017). Antibody-virus co-evolution in HIV infection: paths for HIV vaccine development. Immunol Rev.

[76] Landais E, Huang X, Havenar-Daughton C, Murrell B, Price MA, Wickramasinghe L (2016). Broadly neutralizing antibody responses in a large longitudinal sub-saharan HIV primary infection cohort. PLoS Pathog.

[77] Landais E, Murrell B, Briney B, Murrell S, Rantalainen K, Berndsen ZT (2017). HIV Envelope glycoform heterogeneity and localized diversity govern the initiation and maturation of a V2 apex broadly neutralizing antibody lineage. Immunity.

[78] Moore PL, Gray ES, Wibmer CK, Bhiman JN, Nonyane M, Sheward DJ (2012). Evolution of an HIV glycan–dependent broadly neutralizing antibody epitope through immune escape. Nat Med.

[79] Hraber P, Korber B, Wagh K, Giorgi EE, Bhattacharya T, Gnanakaran S (2015). Longitudinal antigenic sequences and sites from intra-host evolution (LASSIE) identifies immune-selected HIV variants. Viruses.

[80] Tian M, Cheng C, Chen X, Duan H, Cheng HL, Dao M (2016). Induction of HIV neutralizing antibody lineages in mice with diverse precursor repertoires. Cell.

[81] Briney B, Sok D, Jardine JG, Kulp DW, Skog P, Menis S (2016). Tailored immunogens direct affinity maturation toward HIV neutralizing antibodies. Cell.

[82] Jardine J, Julien J-P, Menis S, Ota T, Kalyuzhniy O, McGuire A (2013). Rational HIV immunogen design to target specific germline B cell receptors. Science.

[83] Jardine JG, Ota T, Sok D, Pauthner M, Kulp DW, Kalyuzhniy O (2015). Priming a broadly neutralizing antibody response to HIV-1 using a germline-targeting immunogen. Science.

[84] Sok D, Pauthner M, Briney B, Lee JH, Saye-Francisco KL, Hsueh J (2016). A prominent site of antibody vulnerability on HIV envelope incorporates a motif associated with CCR5 binding and its camouflaging glycans. Immunity.

[85] McGuire AT, Hoot S, Dreyer AM, Lippy A, Stuart A, Cohen KW (2013). Engineering HIV envelope protein to activate germline B cell receptors of broadly neutralizing anti-CD4 binding site antibodies. J Exp Med.

[86] Medina-Ramírez M, Garces F, Escolano A, Skog P, de Taeye SW, del Moral-Sanchez I (2017). Design and crystal structure of a native-like HIV-1 envelope trimer that engages multiple broadly neutralizing antibody precursors in vivo. J Exp Med.

[87] Sliepen K, Medina-Ramírez M, Yasmeen A, Moore JP, Klasse PJ, Sanders RW (2015). Binding of inferred germline precursors of broadly neutralizing HIV-1 antibodies to native-like envelope trimers. Virology.

[88] Kai Xu, Acharya P, Kong R, Cheng C, Chuang G-Y, Liu K (2018). Epitope-based vaccine design yields fusion peptide-directed antibodies that neutralize diverse strains of HIV-1. Nat Med.

[89] Ingale J, Stano A, Guenaga J, Sharma SK, Nemazee D, Zwick MB (2016). High-density array of well-ordered HIV-1 spikes on synthetic liposomal nanoparticles efficiently activate B cells. Cell Rep.

[90] Sliepen K, Ozorowski G, Burger JA, van Montfort T, Stunnenberg M, LaBranche C (2015). Presenting native-like HIV-1 envelope trimers on ferritin nanoparticles improves their immunogenicity. Retrovirology.

[91] Georgiev IS, Joyce MG, Chen RE, Leung K, McKee K, Druz A (2018). Two-component ferritin nanoparticles for multimerization of diverse trimeric antigens. ACS Infect Dis.

[92] McLellan JS, Chen M, Joyce MG, Sastry M, Stewart-Jones GBE, Yang Y (2013). Structure-based design of a fusion glycoprotein vaccine for respiratory syncytial virus. Science.

[93] Krarup A, Truan D, Furmanova-Hollenstein P, Bogaert L, Bouchier P, Bisschop IJM (2015). A highly stable prefusion RSV F vaccine derived from structural analysis of the fusion mechanism. Nat Commun.

[94] Yin HS, Wen X, Paterson RG, Lamb RA, Jardetzky TS (2006). Structure of the parainfluenza virus 5 F protein in its metastable, prefusion conformation. Nature.

[95] Lee JE, Fusco ML, Hessell AJ, Oswald WB, Burton DR, Saphire EO (2008). Structure of the Ebola virus glycoprotein bound to an antibody from a human survivor. Nature.

[96] Pallesen J, Wang N, Corbett KS, Wrapp D, Kirchdoerfer RN, Turner HL (2017). Immunogenicity and structures of a rationally designed prefusion MERS-CoV spike antigen. Proc Natl Acad Sci.

[97] Kong L, Giang E, Nieusma T, Kadam RU, Cogburn KE, Hua Y (2013). Hepatitis C virus E2 envelope glycoprotein core structure. Science.

[98] Hastie KM, Igonet S, Sullivan BM, Legrand P, Zandonatti MA, Robinson JE (2016). Crystal structure of the prefusion surface glycoprotein of the prototypic arenavirus LCMV. Nat Struct Mol Biol.

[99] Zeev-Ben-Mordehai T, Vasishtan D, Hernández Durán A, Vollmer B, White P, Prasad Pandurangan A (2016). Two distinct trimeric conformations of natively membrane-anchored full-length herpes simplex virus 1 glycoprotein B. Proc Natl Acad Sci.

[100] Kirchdoerfer RN, Cottrell CA, Wang N, Pallesen J, Yassine HM, Turner HL (2016). Prefusion structure of a human coronavirus spike protein. Nature.

[101] Zhao Q, Li S, Yu H, Xia N, Modis Y (2013). Virus-like particle-based human vaccines: quality assessment based on structural and functional properties. Trends Biotechnol.

[102] Schrodinger LLC. The PyMOL molecular graphics system, version 1.3r1. 2010.

